# Portable Device for Potentiometric Determination of Antioxidant Capacity

**DOI:** 10.3390/s23187845

**Published:** 2023-09-13

**Authors:** Alla V. Ivanova, Maria G. Markina

**Affiliations:** Chemical Technological Institute, Ural Federal University Named after the First President of Russia B. N. Yeltsin, 19, Mira Str., 620002 Ekaterinburg, Russia; m.g.markina@urfu.ru

**Keywords:** portable device, microcell, potentiometry, antioxidant capacity, screen-printed electrode

## Abstract

For the first time, a prototype of a portable device for the potentiometric determination of antioxidant capacity based on a new measurement principle is proposed. A feature of the approach is the use of an electrochemical microcell with separated spaces and two identical electrodes with immobilized reagents. An antioxidant solution is introduced into one half-cell, and the antioxidants interact with the reagents. The other half-cell contains only reagents. The potential difference between the electrodes is due to the change in the ratio of the oxidized and reduced form of the reagents, which occurs as a result of the reaction with the antioxidants in one of the half-cells and is related to their concentration. The range of linearity of the microcell with immobilized reagents is 40–4000 μM-eq, and the limit of detection is 20 μM-eq. The device was successfully tested in the analysis of standard antioxidant solutions. The recoveries were (92–113)%, and the relative standard deviation did not exceed 15%. A good correlation was found between the data obtained by the approach and the potentiometric method in a macrocell for fruit juice analysis. Pearson’s coefficient for the obtained experimental data was 0.9955. The proposed portable device is promising and can be used in field conditions.

## 1. Introduction

Living organisms constantly produce activated oxygen metabolites—highly reactive, predominantly radical compounds [1]. The activity of free radicals increases dramatically with the adverse effects of the environment (various pollutants, radiation, pathogenic microorganisms). Free radicals can damage lipids in biological membranes [2], membrane-bound proteins [3], enzymes [4], and DNA [5]. At the same time, toxic oxidation products accumulate in the body. The antioxidant defense system resists the damaging effects of free radicals. To describe the imbalance between the process of the formation of activated oxygen metabolites and the protective effect of the antioxidant system, the term “oxidative stress” is used [6,7,8]. A decrease in the activity of antioxidant system components can serve as an indicator of the level of oxidative stress in the body [9]. It has been shown that oxidative stress is involved in the pathogenesis of more than 100 diseases and pathological conditions, and many of them are environment- or age-related pathologies [10,11,12,13,14].

Antioxidants do not behave alone in vivo and act cooperatively in a magnificent network [15]. In this regard, it is important to create approaches to determining the integral parameter that reflects the property of the antioxidant system as a whole—the antioxidant capacity (AOC) [16,17].

Laboratory assessments for estimating the AOC are quite developed, and they include electron paramagnetic resonance [18], optical (chemiluminescent, spectrophotometric, fluorescent) [19,20,21], high-performance liquid chromatography [22,23], and electrochemical methods [24,25]. However, considering the importance of AOC determination and the development of personalized medicine, it is important to create simple portable devices and test systems for express chemical analysis outside the laboratory, without involving expensive equipment and highly qualified specialists. Most approaches to the determination of the AOC in a portable version are based on optical or electrochemical registration of an analytical signal.

Portable optical methods are characterized by low detection limits and high sensitivity [26]. Nevertheless, there are drawbacks of optical devices for determining the AOC: restrictions on the terms and conditions of the storage of test systems [27], the insufficiently narrow specificity of the reagent for the antioxidant (a number of organic and amino acids have an interfering effect on the signal) [28,29], the problem of preserving the spectral characteristics of near-IR spectrometers with a decrease in the size of the device in terms of the range of the wave numbers and resolution, as well as the signal-to-noise ratio [30], technical difficulties in fixing a colorimetric signal in chemosensors using a smartphone camera [31], and the analysis of intensely stained samples.

Electrochemical methods hold promise in solving such problems due to the simplicity of their design and the possibility of creating miniature devices without deteriorating analytical characteristics [32]. Moreover, the interaction of activated oxygen metabolites with antioxidants has an electron–donor–acceptor nature, and electrochemical methods quite closely reflect the system of interaction between radicals and antioxidants in a living organism [33].

There are a few approaches implemented in the form of electrochemical portable devices, test systems that allow AOC determination outside the laboratory [34,35,36,37].

Among the numerous methods for determining the AOC, methods with an integrated approach, reflecting three main mechanisms of antioxidant action in the food/biological matrix, are of undoubted interest: electron transfer reactions from antioxidant to the substrate (ET mechanism), the transfer of a hydrogen atom from an antioxidant to a substrate (HAT mechanism), and the complex formation reaction of antioxidants with metal ions of variable valence [33,38]. However, in the first stage of creating portable devices, ET methods are the most-preferred, because they are not only the easiest to implement in analytical practice, but also quite informative, and the results are in good agreement with conventional methods.

Within the framework of ET methods, the antioxidant effect of the studied samples (model or real objects) is modeled using a reagent—an oxidizing agent or system of oxidizers. Among the systems of model oxidizers (MOs), potassium hexacyanoferrate (III)/(II) has well proven itself due to its advantages such as high electrochemical reversibility, the high rate and stoichiometry of the chemical reaction with the main antioxidants of the objects, and the dependence of the potential of a hexacyanoferrate (III)/(II) pair, which arises from the interaction of the antioxidant with potassium hexacyanoferrate (III); the ratio of the concentrations of the oxidized and reduced reagent forms obeys the Nernst equation, and the value of this potential is established rather quickly and is stable over time. The potassium hexacyanoferrate (III)/(II) system has been tested on a wide range of objects [16,39,40,41,42,43].

However, in portable devices with potentiometric signal detection, the problem of the electrical stability of the reference electrode remains unresolved. The commonly used reference electrode is an Ag/AgCl reference electrode—a silver wire coated with silver chloride. The potential of the electrode is unstable in a reagent medium containing hexacyanoferrate (III/II) ions and a phosphate-buffered saline (PBS) solution. It was shown in [44] that the composition of the precipitate formed in such a medium on the surface of a silver electrode depends on the concentration of the anions in the solution, on the solubility of the resulting silver compounds, and on the potential of the silver electrode. In this case, the formation of several sparingly soluble silver compounds is likely Ag_4_[Fe(CN)_6_], Ag_3_[Fe(CN)_6_], and AgCl at polarization the potentials of the silver wire in the range from 0.05 to 0.2 V and higher. As a result of the formation of a precipitate of a mixed composition on the Ag wire, the potential of such a silver chloride electrode will respond to a change in the concentration of not only chloride ions (Cl^-^), but also hexacyanoferrate (III), (II) anions, demonstrating the instability of the potential. For potentiometric determinations, the stability of the reference electrode is especially important, since even a relatively small potential shift leads to a significant error in the calculation of the results. Thus, a potential shift of 5 mV can lead to an error in AOС determination by 30% or more.

One of the possible ways to solve this problem is to use a device in which the measuring electrode and the reference electrode are made of the same material. In this case, one electrode is immersed in a half-cell with a reagent solution, and the other electrode is immersed in a half-cell with a reagent and an analyte. The half-cells communicate with each other through a semipermeable membrane. The potential difference between the electrodes will depend on the AOC and can be used as an analytical signal. The authors of [45] proposed to use platinum planar, bulk, thick-film, or nanoelectrodes as the electrodes in the laboratory version of the method.

The aim of this work was to create a prototype of a portable device for determining the AOC. The operation of the device is based on the potentiometric method using potassium hexacyanoferrate (III)/(II) as the MO system. The design feature of the microdevice is the presence of two separated spaces, each of which is immersed in one of two identical carbon-containing electrodes with immobilized reagents [46].

## 2. Materials and Methods

### 2.1. Chemicals

The following reagents were used. K_4_[Fe(CN)_6_] and KH_2_PO_4_ were of the purist grade, K_3_[Fe(CN_6_)]; Na_2_HPO_4_·12H_2_O was of a chemically pure grade; hydrochloric acid (ООО Sigma Tek, Moscow, Russia) was of an extra-pure grade. Other chemicals were: L-cysteine, L-ascorbic acid, and reduced L-glutathione, grade ≥ 99.0% (Panreac, Darmstadt, Germany); uric acid, 99% (Sigma-Aldrich, St Louis, MI, USA); and potassium chloride, 99–100.5% (Sigma-Aldrich). Ethanol medical, 95% (OOO Rosbio, St. Petersburg, Russia), acetone (JSC Ekos-1, Moscow, Russia), and acetonitrile (Component-Reaktiv, Moscow, Russia) were of an extra-pure grade.

### 2.2. Materials

Carbon conductive ink Acheson Electrodag PF-407C (Henkel, Rocky Hill, CT, USA) was used for the carbon screen-printed electrode (CSPE) fabrication. STEF-U fiberglass (Type 221, 1С ТC-16-89И79006.002. ТC.) was used as the substrate material.

The CSPE fabrication technique is described in [47]. The carbon ink was applied onto a polymer substrate of 0.035 cm in thickness in the form of strips of 0.2 × 3.8 cm^2^ and with a layer of about a 40 μm thickness. The strips were heat-treated in a drying cabinet in accordance with the regulations of the ink manufacturer and insulated.

Cementit universal (Merz + Benteli AG, Niederwangen, Switzerland) was used as an insulator in the manufacture of the CSPE.

Microcells were made by 3D printing with a Standard Rapid Photopolymer Resin (Longer3D, Shenzhen, China).

### 2.3. Equipment

The acidity of the solutions and potentiometric measurements of the AOC were conducted using a Ta-ion pH meter (Tom’analit, Tomsk, Russia). The DVS-M/1NA (18)-N System (Mediana Filter, Moscow, Russia) for ultrapure deionized water preparation (Ω up to 18 Mom∙cm) was used.

The CSPEs were laboratory-made using a TIC-50B machine for screen printing (China).

Pt wires or the CSPEs were used as the working and reference electrodes. RE-10103 (Ag/AgCl/3 M KCl) (OOO Izmeritelnaya Tekhnika, Moscow, Russia) was used as the reference electrode in the laboratory version of the analysis and pilot experiment.

Stationary models of the microcells were made on the 3D printer Longer Orange 10 (Longer3D, China) from a photopolymer resin that cures at 405 nm.

The dispersity of the immobilized reagent crystals was assessed using a stereomicroscope Soptop szn71 (Sunny Optical Technology Company Limited, Ningbo, China).

The equipment for choosing the reagent immobilization conditions included a Scientz-10N bell-type freeze dryer with the compressor SCOPE (Ningbo Scientz Biotechnology, Ningbo, China); Ultraviolet (UV) lamp Formlabs Form Cure (Formlabs GmbH, Berlin/Heidelberg, Germany); drying cabinet Snol 58/350 (SNOL, Narkūnai, Lithuania); and the drying cabinet ShS-80-01 SPU (Smolensk SKTB SPU, Smolensk, Russia).

### 2.4. Methods and Calculations

The determination of the AOC was based on the previously developed potentiometric method with the MO system [40]. The source of information about the AOC is the potential shift of the working electrode in the system K_3_[Fe(CN)_6_]/K_4_[Fe(CN)_6_], observed when antioxidants (samples) are introduced into the solution. This shift is a consequence of the change in the ratio of the oxidized and reduced forms of the MO system as a result of the reaction:

a∙Fe(III) + b∙АО→a∙Fe(II) + b∙AO_Оx_ (1)

where AO—an antioxidant; AO_Ox_—the oxidation product of the antioxidant; a, b—stoichiometric reaction coefficients; Fe (III)—potassium hexacyanoferrate (III); Fe (II)—potassium hexacyanoferrate (II).

The reaction was carried out in K-Na-phosphate-buffered solution at pH 7.4. The concentrations of K_3_[Fe(CN_6_)]/K_4_[Fe(CN_6_)] in an electrochemical cell were 0.01 mol/L/0.0001 mol/L, respectively. The AOC was calculated as follows [40]:

(2) AOC=COx−αCRed1+α·VcellVal

where α = (C_Ox_/C_Red_)∙10(ΔE)nF/2.3RT; ΔЕ—the EMF of the electrochemical cell, V; C_Ox_—the initial concentration of potassium hexacyanoferrate (III), М; C_Red_—the initial concentration of potassium hexacyanoferrate (II) in the electrochemical cell, М; АОC—concentration of antioxidants in the sample, М-eq, n—number of electrons, participating in the Red/Ox reaction, n = 1; R = 8.31 J·mol^−1^·K^−1^; F = 96,485 C·mol^−1^; T—temperature, K; $\frac{V_{cell}}{V_{al}}$—dilution of the analyte, equal to the ratio of the volume of the microcell space to the volume of the aliquot, ml.

In the experiments with the microcells, CSPEs, and immobilized reagents, the AOC was calculated using the formula:

(3) AOC=COxCRed1−ααCOx+CRed

the designations are the same as in Formula (2).

### 2.5. Microcell Design and Fabrication

The following concept of a portable potentiometric device is proposed. The measuring electrode and the reference electrode were made of the same material—Pt wire or carbon-containing conductive material. The electrochemical microcell of the device consisted of two separated spaces communicating via a semipermeable membrane. A solution of MO (potassium hexacyanoferrate (III)/(II)) of the same composition and an electrode were introduced into each of the half-cells. Then, the antioxidant additive was added into the first half-cell, and the potential difference was recorded. In this case, the measured signal was related to the amount of antioxidants that entered into a chemical reaction with the MO system in the first half-cell (Formula (2)). A variant of a microcell with MO immobilized on the electrode surface was also considered. The use of an immobilized reagent will simplify the analysis procedure and facilitate its implementation in the field.

**Figure 1:**
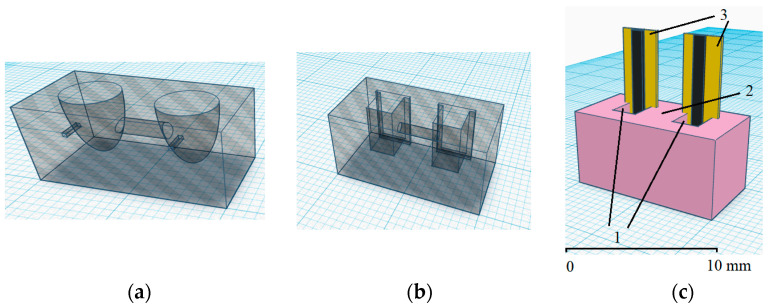
Schematic representation of a microcell with separated spaces for the Pt electrodes (**a**), CSPEs (**b**), and image of the microcell for CSPEs (**c**). Designations: 1—half-cells with a reagent solution and additives of the analyte; 2—semipermeable membrane; 3—CSPEs.

The microcell design was created using a web application (https://www.tinkercad.com/ (accessed on 23 March 2023)). Figure 1 shows the microcell for the Pt wire electrodes (a), planar electrodes—CSPEs—(b), and the appearance of a microcell with separated spaces and the CSPEs (c). The functional diagram of the measuring setup is shown in Figure S1. In the process of developing the microcell design, we relied on the linear dimensions of the Pt electrodes and CSPEs, manufactured in our laboratory [47,48]. The shape of the half-cells was matched with the shape of the electrodes used: for planar CSPEs, the spaces were made in the form of rectangular parallelepipeds and, for Pt wire electrodes, in the form of hemispheres. The volume of each of the two half-cells was 100 µL.

The microcells were 3D printed from polyacrylate-based photopolymer resin cured at 405 nm.

An asbestos filament impregnated with 0.1 M KCl solution was used as the material for the half-cell’s connection.

**Figure 2:**
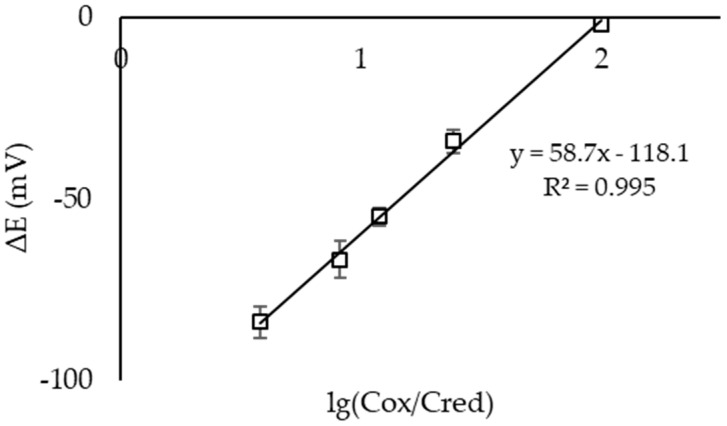
Dependence of the EMF of an electrochemical microcell with Pt electrodes on the logarithm of the С(K_3_[Fe(CN)_6_])/С(K_4_[Fe(CN)_6_]) ratio in solution in the first half-cell. Background electrolyte: 0.1 M KCl, 0.005 M PBS, pH 7.4 (n = 3, P = 0.95).

Figure 2 shows the dependence of the EMF of an electrochemical microcell with Pt electrodes on the logarithm of the ratio of the hexacyanoferrate (III)/(II) ions’ concentrations in the first half-cell when ready-made solutions of MO were used. The slope of the obtained dependencies was close to the theoretical one (59.17 mV at 25 °C).

### 2.6. Reagents’ Immobilization on CSPEs

The concentrates of the reagent mixture solution (n = 20-; 10-; 5-fold), having the following composition: (n*0.01)/(n*0.0001) M/M solution of potassium hexacyanoferrate (III)/(II), (n*0.005) M PBS, and (n*0.1) M KCl solution, were prepared. A drop of 5 μL of 20-fold concentrate (10 μL of 10-fold concentrate, 20 μL of 5-fold concentrate) was applied to the CSPE work surface. The drying of the reagent drops on the electrodes was carried out in a drying cabinet at 50 °C for 15 min. Then, the electrodes were kept at room temperature for 30 min and then used in the analysis.

### 2.7. Microcells’ Testing

**Figure 3:**
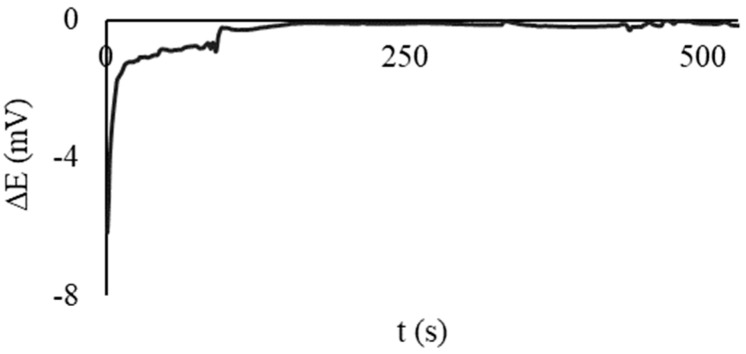
Time dependence of electrochemical microcell EMF for CSPEs immersed in half-cells with a solution of 10^−2^/10^−4^ M potassium hexacyanoferrate (III)/(II). Background electrolyte: 0.1 M KCl, 0.005 M PBS, pH 7.4.

The microcells’ testing before the measurements was performed using ready-made solutions of 10^−2^/10^−4^ M potassium hexacyanoferrate (III)/(II) and two CSPEs. The CSPEs were placed in the half-cells, and 100 µL of the MO solution was added there. An example of the obtained microcell EMF-time dependence is shown in Figure 3. If the steady value of the EMF was close to 0 V, it was assumed that the microcell was ready for operation.

**Figure 4:**
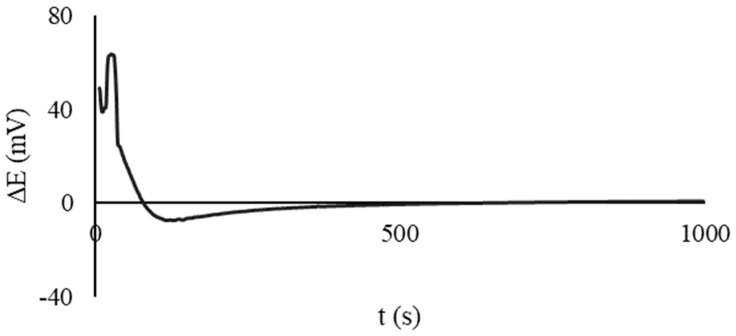
Time dependence of the EMF of an electrochemical microcell for CSPEs with immobilized reagents; the composition of the solution in the half-cells after dissolution of the reagents is 10^−2^/10^−4^ M potassium hexacyanoferrate (III)/(II), 0.1 M KCl, 0.005 M PBS, pH 7.4.

The preliminary testing of the microcells with the CSPEs and immobilized reagents was performed in a similar manner. Both half-cells were filled with CSPEs, and 100 µL of deionized water was added. The EMF was recorded over time. An example of the obtained dependence is shown in Figure 4. If the steady value of the EMF was close to 0 V, it was considered that the microcell was ready for operation.

### 2.8. AOC Determination Using Microcells

#### 2.8.1. AOC Determination with a Ready-Made Solution of Reagents

An aqueous solution containing 0.1 M KCl, 0.005 M phosphate buffer, pH = 7.4, and 10^−2^/10^−4^ М K_3_[Fe(CN)_6_]/K_4_[Fe(CN)_6_] was added to each of the two half-cells in a volume of 90 μL. Two Pt wires or two CSPEs were used as the reference and working electrodes. An aliquot of 10 µL of the antioxidant model solution was added to one half-cell. The EMF value of the electrochemical microcell was recorded. The steady value of the EMF was used to calculate the AOC of the analyzed solution (Formula (2)).

#### 2.8.2. AOC Determination with Immobilized Reagents

One-hundred microliters of deionized water was added to one half-cell, and the same volume of the analyzed solution (antioxidant model solution or sample) was added to the other half-cell. Two CSPEs with immobilized reagents were used as the reference and working electrode. The EMF value of the electrochemical microcell was recorded. The EMF value after signal stabilization was used to calculate the AOC of the sample (Formula (3)).

### 2.9. Statistical Analysis

The measurements were performed in 3–5-fold replication (4–5 times in the model conditions and 3 in the real conditions). Statistical analysis was performed in Microsoft Excel 2010 with an accepted confidence level (P) of 0.95. The data are presented as X ± ∆X, where X is the mean value and ∆X is the confidence interval. The correctness of the AOC determination by the developed technique was evaluated by the spike recovery test with the use of the model solutions of non-enzymatic antioxidants and their mixtures present in the object of analysis. The recovery of the antioxidants was determined in accordance with the IUPAC recommendations [49]. The relative standard deviation (RSD) was used to determine the reproducibility of the measurement results. The validation of the results of the AOC determination of the samples obtained on the developed device was performed in relation to the results obtained by potentiometry in the macrocell, based on the F- and *t*-tests. The correlation analysis was performed by calculating the Pearson correlation coefficient.

## 3. Results and Discussion

### 3.1. Selection of Working Conditions of the Analysis

#### 3.1.1. Electrode Material

In the classical version of the potentiometric method for AOC determination, Pt-containing electrodes, including Pt thick-film screen-printed electrodes [40,50], are used as the working ones [16]. However, in the case of portable devices and test systems, which are supposed to be disposable in the future, the use of platinum-based electrodes leads to an increase in the cost of analysis and seems inappropriate. Carbon-containing printed electrodes are considered as electrode materials in the development of a portable micro-device.

The indicator electrode should be adsorption and chemically and electrochemically stable in the range of the operating potentials of the model oxidizer and should also provide a fast exchange of electrons between its oxidized and reduced forms [51]. The source of information about these processes can be potentiograms showing the rate of stabilization of the electrode potential in the MO solution and the slope of the dependence of the electrode potential on the logarithm of the ratio of the oxidized and reduced forms of MO.

**Figure 5:**
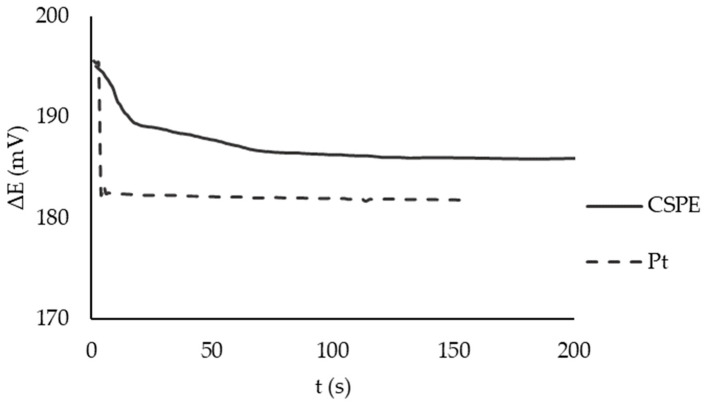
Time dependence for electrodes potential: Pt (dashed line) and CSPE (solid line) in the macrocell without stirring. The reagent solution contained 10^−2^/10^−4^ М potassium hexacyanoferrate (III)/(II), 0.1 М KCl, 0.005 М PBS, pH 7.4, T = 25 °C, Ag/AgCl/3 M KCl reference electrode.

CSPEs are slightly inferior to Pt electrodes in terms of the potential stabilization time in MO solution (Figure 5). This is probably due to the high resistance of the carbon material itself; in addition, the components of the conductive ink slow down the heterogeneous charge transfer kinetics on the CSPEs’ surface, such as polymer binders, resins, or cellulose acetate, solvents (terpineol, ethylene glycol, cyclohexanone), and various functional additives [52,53]. In this regard, a number of authors propose to activate the electrodes to increase the charge transfer rate, as a rule, by electrochemical methods [54,55,56,57,58,59]. However, as was shown in [60,61], an increase in the catalytic activity of the electrode worsens the measurements of the redox potential.

**Figure 6:**
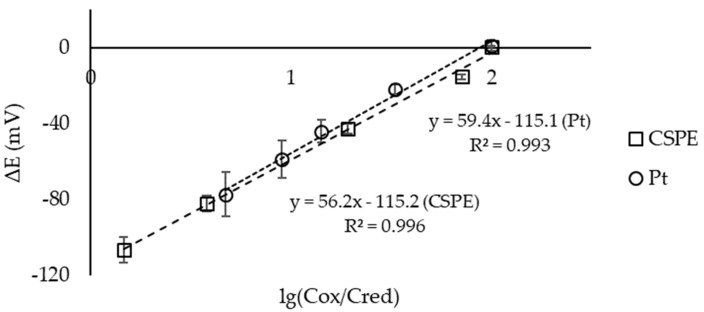
Dependence of the potential of the Pt electrodes (o) and CSPEs (□) on the logarithm of the ratio C(K_3_[Fe(CN)_6_])/C(K_4_[Fe(CN)_6_]) in solution in the first half-cell. Background electrolyte: 0.1 М KCl, 0.005 М PBS, pH 7.4 (n = 3, P = 0.95).

Figure 6 shows the dependence of the EMF of a microcell (Pt electrodes, CSPEs) on the logarithm of the concentration ratio K_3_[Fe(CN)_6_]/K_4_[Fe(CN)_6_] in the first half-cell. The experiment used a microcell with separated spaces and two identical electrodes. It can be seen that the slopes of the dependencies for both the Pt electrodes and CSPEs were close to the theoretical slope of 59.17 mV/decade at 25 °C.

Thus, electrodes based on conductive carbon-containing materials are alternative candidates for replacing Pt electrodes [62], and their use as working electrodes opens the prospect for the creation of portable potentiometric disposable devices.

#### 3.1.2. Selection of Reagents’ Solution Composition for Immobilization on CSPEs

In this work, it is proposed to use a ready-made mixture of reagents, which will be applied to the electrodes, dried, and, during analysis, quickly dissolved in water and the analyzed solution. When developing the composition of the applied reagents’ solution, these requirements were followed:

- The reagents’ solution obtained after the dissolution of the precipitate must provide physiological conditions for the analysis. Since antioxidants can contain H^+^ ions and change the pH of the medium during analysis, so the use of a buffer solution is necessary. In addition, the acidity of the medium affects the potential of the working electrode. Therefore, to obtain stable results, it is necessary to create a buffer medium that would prevent the potential shift that is not caused by the reaction of an antioxidant with a model oxidant. At рН = 7.4, the reaction rate between the antioxidant and К_3_[Fe(CN)_6_] is rather high [63], and the capacity of the K-Na-phosphate-buffered solution (PBS) is sufficient to compensate for the pH shift when a sample is added to the cell [64,65].
- The reagent solution must have sufficient electrical conductivity to implement potentiometric measurements.
- The immobilized reagent should be uniform, finely dispersed, and quickly dissolve in a small volume of water and the analyzed solution.

Considering Requirement (c), in order to obtain the most-uniform, finely dispersed crystals on the electrodes, it is advisable to reduce the concentration of the components of the reagents’ solution to the minimum possible, while observing the first two requirements. Further research was aimed at finding such a composition of the reagents’ solution.

- Buffer solution:

The results of calculating the minimum buffer concentration required to maintain the pH of the analyzed solution in the physiological range pH = (7.4 ± 0.1) when adding 0.001 M ascorbic acid to it are shown in Table 1. The concentration of the ascorbic acid additive chosen for the calculations as a model antioxidant corresponds to the maximum AOC level of a wide range of potential objects [40,66]. The calculations showed that a PBS concentration of 0.005 M satisfies the requirement.

- Background electrolyte:

When selecting the minimum buffer concentration, the required level of electrical conductivity of the reagents’ solution is not achieved. This can be compensated by adding the supporting electrolyte KCl. Potassium chloride is a biocompatible compound, and the values of the mobility of K^+^ and Cl^−^ ions are close to and greater than those for the phosphate ions of the PBS; therefore, it is possible to achieve the required electrical conductivity of the solution by adding a smaller amount of salt.

The minimum concentration of potassium chloride was selected experimentally. Table 2 shows the linear regression equations for the dependences of the CSPE potential on the logarithm of the K_3_[Fe(CN)_6_]/K_4_[Fe(CN)_6_] concentration ratio at different concentrations of KCl in solution in a macrocell. The results demonstrated that the slope of the linear regressions was close to the theoretical one over the entire studied range of KCl concentrations. The smallest scatter of potential values was observed for C(KCl) ≥ 0.1 M, and this concentration was chosen as the working one.

#### 3.1.3. Selection of a Method and Conditions for Reagents’ Immobilization on CSPEs

On the way to creating a prototype of a portable microdevice for the potentiometric determination of the AOC, the main task was to select the deposition method and conditions for the immobilization of reagents on planar electrodes (CSPEs). As a result of the study of [67,68,69,70] and evaluating the advantages and difficulties of implementing technically complex methods, the drop-cast method of applying and crystallizing reagents from a drop of an evaporating solution was chosen.

In the case of the dropwise application of reagents to obtain a finely dispersed precipitate, according to the literature data, it is necessary to influence the rate of solvent evaporation and/or the initial drop height. The last of the listed parameters was the most difficult to vary, since the materials of the substrate and the CSPE conductive layer are hydrophobic. The ability to influence the drop height of the reagent solution by using organic or water–organic mixtures is also limited by the low solubility of potassium hexacyanoferrates (II, III) in the most-commonly used organic solvents (ethanol, acetonitrile, acetone). The limited working surface area of the electrodes forces the use of reagent solution concentrates and, consequently, small drop volumes of the concentrated reagents’ solutions.

In this regard, 20-, 10-, or 5-fold reagents’ solution concentrates were prepared as the initial concentrates. The immobilization of the reagents on the CSPEs was carried out according to the method described in Section 2.6. More dilute concentrates were not prepared because droplets larger than 20 µL spilled out of the CSPE substrate boundaries. The preparation of more-concentrated solutions is limited by the solubility of the components of the reagent mixture.

The intensity of solvent evaporation can be influenced by heating, convection, or cooling, followed by sublimation of the solvent. The following conditions for the reagents’ immobilization on the electrode surface were considered: drying under laboratory conditions (at room temperature, atmospheric pressure, and relative air humidity of 25–35%), freeze-drying (rapid cooling to −30 °С, sublimation), drying by heating in an oven with and without forced convection, and UV drying.

The heat treatment conditions were selected based on the literature data on the thermal stability of the components of the MO mixture, the background solution, the substrate material, and the conductive CSPE layer. It is known that the decomposition temperatures of almost all components of the mixture are quite high (T_melt_(KCl) = 776 °C, T_melt_(KH_2_PO_4_) = 252.6 °С, T_melt_(Na_2_HPO_4_) = 250 °С, T_melt_(K_3_[Fe(CN)_6_]) over 300 °С), with the exception of potassium hexacyanoferrate (II) (T_melt_ = 69 °С) [71]. The recommended drying temperature for carbon ink is in the range of 90–120 °С. The permissible operating temperature range of the fiberglass substrate is from −65 to 155 °С. Thus, the temperature chosen for the experiments was 50 °С.

Drying in an oven without convection was carried out for 60 min, as with convection. It has been experimentally determined that 15–20 min are sufficient when drying in a cabinet with convection.

Table S1 shows the EMF values of the microcells with the immobilized reagents on the CSPEs and the microcells with a ready-made solution of reagents for different immobilization conditions. The dissolution rate of the immobilized reagents on the CSPEs was estimated from the EMF-t potentiogram recorded in a microcell. At this stage of the study, to assess the correctness of the proposed approach, a standard silver chloride electrode immersed in a 0.1 M KCl solution in the second half-cell was used as a reference electrode. The steady value of the EMF of the microcells with the reagents immobilized on the CSPEs was compared with the expected value (EMF of a microcell with a prepared solution of reagents of the same composition), and the reproducibility of this value was evaluated.

The freeze-drying method produced a bulk porous mass of reagents, which easily broke off during any manipulations with the electrode, while signal reproducibility deteriorated. No advantages were observed in terms of the rate of dissolution of the reagents’ crystals. The patterns of drops dried by other methods turned out to be typical; the crystals grew mainly along the perimeter of the drops. This is explained by the fact that the emerging centers of crystallization in the process of the drop evaporation were displaced due to capillary forces to the edge of the drop (the effect of coffee rings [72]). However, drying a drop of reagents at room temperature gave larger crystals; this was rather long (over 1 h); the control and fixation of parameters such as the temperature, humidity, and convection is limited, which can affect the dispersity of the crystals and, consequently, reproducibility of the signal. In general, other drying methods with the studied heating methods gave similar results.

Thus, the drop-cast method was chosen for applying a concentrate of the reagents’ solution on the electrodes and immobilization when heated to 50 °С with convection, 15 min. This method gave fast and reproducible results. The volume of the concentrated solution drop (5 µL) ensured the safety of the immobilized reagents during any manipulations and work with the electrodes.

### 3.2. Analytical Characterization

**Figure 7:**
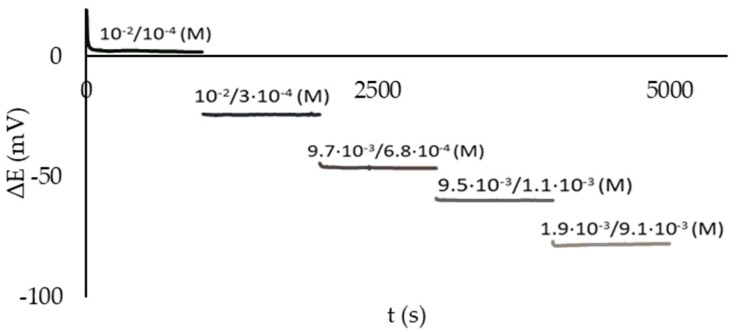
Time dependence of the EMF of an electrochemical microcell using CSPEs for different C(К_3_[Fe(CN)_6_])/C(K_4_[Fe(CN)_6_]) ratios in the first half-cell. Background electrolyte: 0.1 M KCl, 0.005 M PBS, pH 7.4.

The performance of the developed approach to the potentiometric determination of the AOC in the pilot experiments was evaluated as follows. Both spaces of the microcell were filled with MO solution. Various additives of potassium hexacyanoferrate (II) or standard solutions of antioxidants were introduced into the first half-cell, thus varying the ratio K_3_[Fe(CN)_6_]/K_4_[Fe(CN)_6_] in the half-cell. The composition of the solution in the second half-cell was not changed during the experiment. The potential difference between the CSPEs immersed in the half-cells was related to the concentration of the antioxidant entered in the first half-cell. Figure 7 shows the dependence of the EMF of an electrochemical microcell on time when using CSPEs for different concentration ratios K_3_[Fe(CN)_6_]/K_4_[Fe(CN)_6_] in the first half-cell (the concentration ratios are shown in the graph). The dependence demonstrated stable values of the CSPE potentials (up to 1000 s) in the investigated range of antioxidant concentrations.

**Figure 8:**
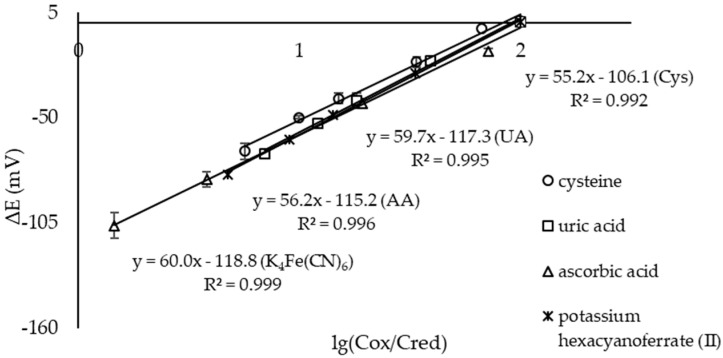
Dependence of the EMF of an electrochemical microcell with CSPEs on the logarithm of the C(K_3_[Fe(CN)_6_])/C(K_4_[Fe(CN)_6_]) ratio in solution when standard solutions were added to the first half-cell: o—cysteine (Cys), □—uric acid (UA), Δ—ascorbic acid (AA), ж—potassium hexacyanoferrate (II). Background electrolyte: 0.1 М KCl, 0.005 М PBS, pH 7.4 (n = 5, P = 0.95).

The slopes of the Nernstian dependences of the electrochemical microcell with the CSPEs and ready-made solutions of reagents when standard solutions of antioxidants were added were close to the theoretical 59.17 mV/decade at 25 °C (Figure 8).

**Figure 9:**
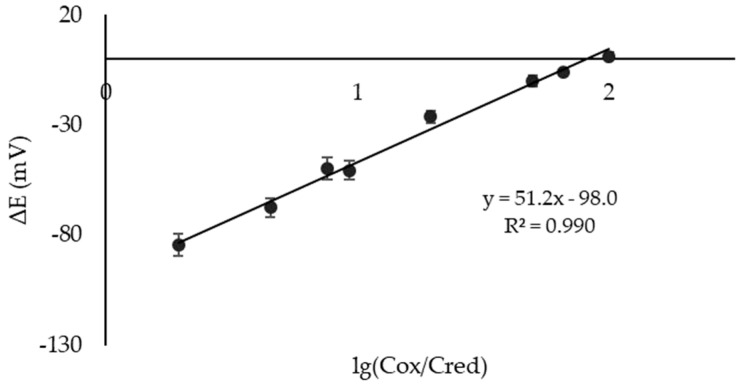
Calibration dependence (E − lg(Cox/Cred) for an electrochemical microcell with CSPEs and immobilized reagents (n = 5, P = 0.95).

Then, similar dependencies were recorded for a microcell with the immobilized reagents on the CSPEs. The range of the AOC values for an electrochemical microcell with the CSPEs and immobilized reagents was 40–4000 µM-eq; the detection limit was 20 µM-eq; the calibration dependence equation was Е = 51.2·lg(Cox/Cred) − 98.0, R^2^ = 0.990 (Figure 9).

The correctness of the quantitative determination of the AOC by the developed method using a microcell and the CSPEs was evaluated by the spike recovery test with the use of model solutions of non-enzymatic antioxidants and their mixtures (Table 3 and Table 4). The recoveries were 85–114% (ready-made solution of reagents) and 92–113 (immobilized reagents on the CSPEs), and the RSD value did not exceed 8% for the solutions and 15% when using the immobilized reagents.

The reasonable recovery values (Table 3 and Table 4) demonstrated the possibility of using the portable device using immobilized reagents in real objects for AOC determination.

### 3.3. Sample Analysis

The developed approach was used to determine the AOC of fruit juices for children produced industrially (Table 5). The potentiometric method using the K_3_[Fe(CN)_6_]/K_4_[Fe(CN)_6_] system (laboratory version using the macrocells) was used as a reference [40].

The value of the calculated Pearson’s coefficient for the obtained experimental data was 0.9955. Since the value was close to 1, this indicated a high positive correlation between the data obtained by the proposed and the reference method.

## 4. Conclusions

A prototype of a portable device for the potentiometric determination of the AOC was developed. The working unit of the device is an electrochemical microcell with separated spaces and two identical electrodes. The spaces communicate through a semi-permeable membrane. The operating conditions of the analysis were selected, and the material of the electrodes, the composition of the reagents’ solution, and the method and conditions for the reagents’ immobilization on the electrode surface were justified. Two identical printed electrodes (CSPEs) were used as the working and reference electrodes. The reagents’ immobilization on the CSPEs was carried out by the drop-casting method for a concentrated solution of the reagents onto the electrodes and holding for 15 min at 50 °С with convection. The microcell with the CSPEs and immobilized reagents gave a potentiometric response with calibration dependence E = 51.2·lg(Cox/Cred) − 98.0 in an AOC range of 40–4000 μM-eq with an LOD of 20 μM-eq. The proposed microcell was applied to determine the AOC of the model solutions of antioxidants and fruit juices, and the recoveries were 85–114% (prepared reagent solution) and 92–113% (immobilized reagents). The RSD value for the reagent solution was no more than 8%; for the immobilized reagents, it did not exceed 15%. The laboratory version of the potentiometric method of analysis using a macrocell served as a reference in determining the AOC of the juices. The results obtained in the macrocells and microcells were correlated (Pearson’s coefficient of 0.9955). The use of a microcell with separated spaces and two identical electrodes with immobilized reagents is promising for the out-of-lab determination of the AOC. It could be easily adapted as a single-use device for on-site analysis in the future.

## 5. Patents

The work on the creation of a microcell with separated spaces formed the basis of the application for invention 2023112943 “A method for determining the antioxidant capacity of substances and a device for its implementation”. Authors: A.V. Ivanova, M.G. Markina, E.L. Gerasimova, and V.I. Kirillova. Priority date: 19 May 2023.

## Figures and Tables

**Table 1:** Buffer capacity and pH of the PBS at different ratios of buffer components and the ascorbic acid additive (initial PBS рН of 7.4).

С(Na_2_HPO_4_), M	С(KH_2_PO_4_), M	Buffer Capacity PBS (pH 7.4), М	pH PBS after 0.001 М Ascorbic Acid Addition
0.0006	0.0004	0.001	6.94
0.0012	0.0008	0.001	7.17
0.0018	0.0012	0.002	7.25
0.0024	0.0016	0.002	7.28
0.0030	0.0020	0.003	7.31
0.0037	0.0023	0.003	7.32
0.0043	0.0027	0.004	7.33
0.0049	0.0031	0.004	7.34
0.0055	0.0035	0.005	7.35
0.0061	0.0039	0.005	7.35

**Table 2:** Linear regression equations for the dependence of the CSPE potential on the logarithm of the ratio C(K_3_[Fe(CN)_6_])/C(K_4_[Fe(CN)_6_]) in solution at background electrolyte concentration of KCl of (0–1.00) М (n = 3, P = 0.95).

С(KCl), M	Linear Regression Equation (E-lg(Cox/Cred)	R^2^
0.00	y = (61.9 ± 1.8)x + (189.1 ± 0.2)	0.9987
0.02	y = (58.5 ± 2.0)x + (198.2 ± 1.6)	0.9998
0.05	y = (59.1 ± 1.0)x + (205.6 ± 0.6)	0.9999
0.10	y = (59.3 ± 0.3)x + (214.5 ± 0.8)	0.9999
0.20	y = (59.4 ± 0.4)x + (226.4 ± 0.2)	0.9999
0.50	y = (59.2 ± 1.3)x + (246.3 ± 0.8)	0.9999
1.00	y = (59.5 ± 0.5)x + (263.7 ± 0.3)	0.9999

**Table 3:** Potentiometric determination of model solutions’ AOС in a microcell with ready-made solutions of the reagents (n = 5, P = 0.95).

Antioxidant	Introduced, ·10^−5^ mol-eq/L	Found, ·10^−5^ mol-eq/L	Recovery, %	RSD ^1^, %
ascorbic acid	4.00	3.96 ± 0.24	99	5
	20.0	20.2 ± 1.2	101	5
	100	93.6 ± 6.0	94	5
	200	190.0 ± 1.5	95	7
uric acid	73.5	62.7 ± 6.2	85	8
	135	118 ± 11	88	8
mixes:				
cysteine:ascorbic acid = 1:1	5.50	5.70 ± 0.40	103	5
cysteine:ascorbic acid = 2:1	29.1	33.3 ± 2.9	114	8
cysteine:ascorbic acid = 1:3	38.5	41.9 ± 3.1	109	7

^1^ Relative standard deviation.

**Table 4:** Potentiometric determination of model solutions’ AOС in a microcell using the immobilized reagents (n = 4, P = 0.95).

Antioxidant	Introduced, ·10^−4^ mol-eq/L	Found, ·10^−4^ mol-eq/L	Recovery, %	RSD ^1^, %
ascorbic acid	17.3	19.6 ± 3.5	113	14
	33.3	33.5 ± 3.2	101	11
gallic acid	1.6	1.5 ± 0.4	93	15
	3.1	2.8 ± 0.8	92	15
chlorogenic acid	1.0	1.0 ± 0.2	104	14
pyrogallol	3.0	3.0 ± 0.2	102	6
mixes:				
cysteine:ascorbic acid = 1:1	2.2	2.0 ± 0.3	93	6
cysteine:ascorbic acid = 1:3	4.4	4.2 ± 0.2	96	2

^1^ Relative standard deviation.

**Table 5:** Results of the potentiometric determination of the AOС of fruit juices in a microcell using the immobilized reagents and laboratory potentiometric method as a reference (n = 3, P = 0.95).

Object of Analysis	АОC (Microcell), mmol-eq/L	RSD ^1^, %	АОC (Macrocell), mmol-eq/L	RSD, %	F ^2^	*t* ^3^
1. Berry mix juice (Frutonyanya)	0.31 ± 0.04	5.8	0.28 ± 0.02	3.8	5.3	0.01
2. Rosehip–apple juice (Agusha)	1.68 ± 0.10	2.3	1.88 ± 0.06	1.4	2.3	0.04
3. Apple–cherry juice (Sady Pridonya)	0.32 ± 0.06	7.2	0.55 ± 0.07	5.7	0.8	0.05
4. Apple–grape juice (Dary Kuban)	0.15 ± 0.02	12.5	0.12 ± 0.02	8.1	3.5	0.01
5. Rosehip–apple juice (Dary Kuban)	2.17 ± 0.15	5.6	2.56 ± 0.13	2.1	5.3	0.04
6. Apple juice (Agusha)	0.19 ± 0.01	2.4	0.17 ± 0.02	3.4	0.6	0.01

^1^ Relative standard deviation; ^2^ Fisher’s test (F_teor_ = 19.0 for f_1_ = n_1_ − 1 = 2, f_2_ = n_2_ − 1 = 2, and P = 0.95); ^3^ Student’s test (t_teor_ = 3.188 for f = n_1_ + n_2_ − 2 = 4 and P = 0.95).

## Data Availability

The data are contained within the article and the Supplementary Material.

## Supplementary Materials

The following Supporting Information can be downloaded at: https://www.mdpi.com/article/10.3390/s23187845/s1, Figure S1: The functional diagram of the measuring setup, Table S1: EMF of the microcells with immobilized reagents on the CSPEs and a ready-made solution of reagents for different immobilization conditions and the corresponding micrographs of the CSPEs (n = 5, P = 0.95).
