# Supplementary material for: Portable Device for Potentiometric Determination of Antioxidant Capacity
Source: Sensors (Basel). 2023 Sep 13;23(18):7845. doi: 10.3390/s23187845 (PMC10536404; doi:10.3390/s23187845)
Supplement: Supplementary file 1 [file sensors-23-07845-s001.zip › sensors-2530704-supplementary.pdf]

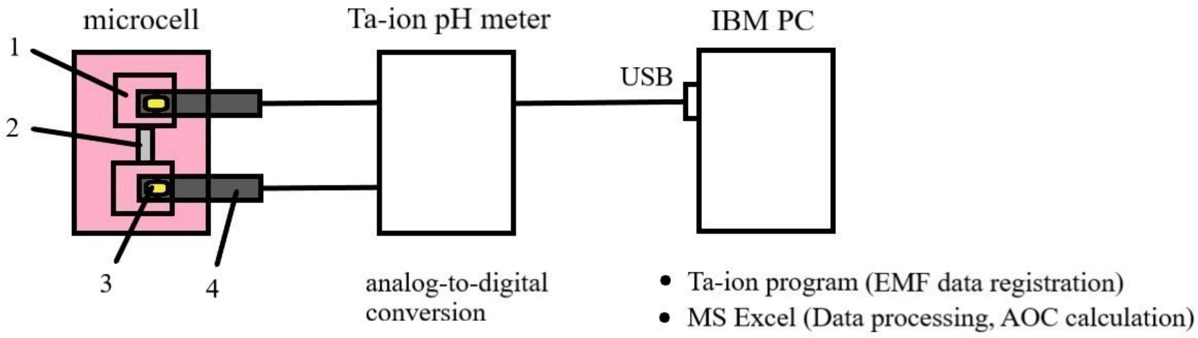

Figure S1.The functional diagram of the measuring setup. Designations: 1 - half-cell, 2 - semipermeable membrane, 3 - immobilized reagent, 4 – CSPE.

Table S1. EMF of microcells with immobilized reagents on CSPE and a ready-made solution of reagents for different immobilization conditions and corresponding micrographs of CSPE (n=5, P=0.95)

Reagents immobilization conditions	(EMFaverage±Δ), mV (microcell with immobilized reagents)	(EMFaverage±Δ), mV (microcell with a ready solution of reagents)	Micrograph of electrodes with immobilized reagents (2.5-fold magnification)
freeze drying	333±71	339 ± 5	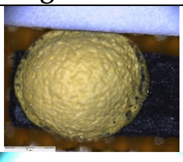
keeping at room temperature	320±3		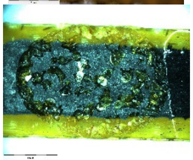
heating (drying cabinet)	336±9		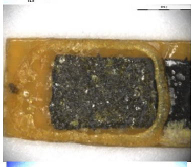
heating with convection (drying cabinet)	338 ± 4		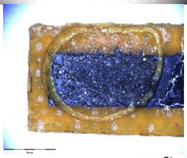
UV-drying	313 ± 9		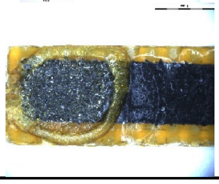
